# Gender‐specific estimates of sleep problems during the COVID‐19 pandemic: Systematic review and meta‐analysis

**DOI:** 10.1111/jsr.13432

**Published:** 2021-07-09

**Authors:** Zainab Alimoradi, David Gozal, Hector W. H. Tsang, Chung‐Ying Lin, Anders Broström, Maurice M. Ohayon, Amir H. Pakpour

**Affiliations:** ^1^ Social Determinants of Health Research Center Research Institute for Prevention of Non‐Communicable Diseases Qazvin University of Medical Sciences Qazvin Iran; ^2^ Department of Child Health and the Child Health Research Institute The University of Missouri School of Medicine Columbia MO USA; ^3^ Department of Rehabilitation Sciences The Hong Kong Polytechnic University Kowloon Hong Kong; ^4^ Institute of Allied Health Sciences National Cheng Kung University Hospital College of Medicine National Cheng Kung University Tainan Taiwan; ^5^ Department of Nursing School of Health and Welfare Jönköping University Jönköping Sweden; ^6^ Stanford Sleep Epidemiology Research Center (SSERC) School of Medicine Stanford University Palo Alto CA USA

**Keywords:** COVID‐19, gender, insomnia, prevalence, sleep

## Abstract

The outbreak of the novel coronavirus disease 2019 (COVID‐19) changed lifestyles worldwide and subsequently induced individuals’ sleep problems. Sleep problems have been demonstrated by scattered evidence among the current literature on COVID‐19; however, little is known regarding the synthesised prevalence of sleep problems (i.e. insomnia symptoms and poor sleep quality) for males and females separately. The present systematic review and meta‐analysis aimed to answer the important question regarding prevalence of sleep problems during the COVID‐19 outbreak period between genders. Using the Preferred Reporting Items for Systematic Reviews and Meta‐Analyses guideline and Newcastle–Ottawa Scale checklist, relevant studies with satisfactory methodological quality searched for in five academic databases (Scopus, PubMed Central, ProQuest, Web of Science , and EMBASE) were included and analysed. The protocol of the project was registered in the International Prospective Register of Systematic Reviews (PROSPERO; identification code CRD42020181644). A total of 54 papers (*N* = 67,722) in the female subgroup and 45 papers (*N* = 45,718) in the male subgroup were pooled in the meta‐analysis. The corrected pooled estimated prevalence of sleep problems was 24% (95% confidence interval [CI] 19%–29%) for female participants and 27% (95% CI 24%–30%) for male participants. Although in both gender subgroups, patients with COVID‐19, health professionals and general population showed the highest prevalence of sleep problems, it did not reach statistical significance. Based on multivariable meta‐regression, both gender groups had higher prevalence of sleep problems during the lockdown period. Therefore, healthcare providers should pay attention to the sleep problems and take appropriate preventive action.

## 1 INTRODUCTION

The outbreak of the novel coronavirus disease 2019 (COVID‐19) changed most people’s lifestyles globally. Indeed, many countries and governments used different policies (e.g. city lockdown, boarder control, online teaching, and special distancing) to slow down the COVID‐19 infection rate (Chen et al., 2020; Chen, Chen et al., 2021); as COVID‐19 was found to have an extraordinary transmission rate and cause an alarming number of deaths (Ahorsu, Lin, Imani et al., 2020; Mamun et al., 2021). With the high prevalence and level of mortality (WHO, 2020), COVID‐19 has impacted peoples psychological health. Indeed, numerous studies have found that COVID‐19 together with the reactions toward controlling COVID‐19 infection are associated with different aspects of psychological health, including depression, anxiety, stress, and sleep problems (Ahorsu, Lin, & Pakpour, 2020; Chang et al., 2020; Lin, Broström et al., 2020, Lin, Imani et al., 2020).

Among the psychological health aspects, sleep is one of the major concerns for healthcare providers (Pakpour et al., 2020) for the following reasons. First, sleep is an essential component for individuals having effective cognitive and emotional processing, and a good night’s sleep is proposed to be vital for all people (Garbarino et al., 2016; Kopasz et al., 2010; Tarokh et al., 2016; Yaffe et al., 2014). Second, ample evidence has shown that sleep is a key factor for individuals maintaining satisfactory and good health, including physical functioning, mental functioning, social functioning, spiritual functioning, and overall quality of life (Garbarino et al., 2016; Gradisar et al., 2008; Shochat et al., 2014). Third, an association between good sleep and health behaviours have been proposed (Lin, Strong et al., 2018, Lin, Lin et al., 2018). However, individuals living in the modern world have different obstacles for achieving good sleep (Strong et al., 2018), given that the technology today contributes to sleep disturbance (Alimoradi et al., 2019). Moreover, recent research shows that problematic social media use, a behaviour found to have increased during the COVID‐19 outbreak (Hashemi et al., 2020; Lin, Broström et al., 2020), is associated with poor sleep (Wong et al., 2020). In short, there is a need to investigate in‐depth the sleep problems occurring during the COVID‐19 outbreak period.

The available literature on COVID‐19 shows the findings of sleep problems. Zhang, Zhang et al. (2020) studied sleep problems amongst healthcare workers and found different prevalence rates of insomnia between non‐medical healthcare workers (e.g. volunteers in the hospital, medical students, and community workers; prevalence of 38.4%) and medical healthcare workers (e.g. medical doctors and nurses; prevalence of 30.5%). Wang, Song et al. (2020) also examined sleep problems in four populations and found different prevalence rates as well. The prevalence of sleep problems among medical staff was 66.1%, in non‐medical staff was 47.8%, in frontline healthcare providers was 68.1%, and in non‐frontline healthcare providers was 64.5%. Although the information on sleep problems during the COVID‐19 outbreak period has been studied and reported, healthcare providers need synthesised information regarding sleep problems across gender. However, to the best of the present authors’ knowledge, no empirical studies have focussed on the sleep problems between genders during the COVID‐19 pandemic, although the studies have controlled for gender in their statistical analyses.

Gender is an important issue for sleep because different treatments may be designed or used for different genders. More specifically, prior evidence has shown that males and females have different processes in brain functions (Xin et al., 2019). Therefore, males and females may not always share the same values on everything. For example, prior research indicates that males as compared with females appreciate physical activity more (Ou et al., 2017). Additionally, males and females report different levels of psychological health (including quality of life) from children and older people (Lin et al., 2016; Su et al., 2013). Therefore, it is important for healthcare providers to understand sleep problems separately for males and females during the COVID‐19 outbreak period.

To answer the important question regarding prevalence of sleep problems during the COVID‐19 outbreak period across gender, the present study was designed and conducted as a systematic review and meta‐analysis. With the robust methods used in the present review, information on sleep problems across gender were synthesised and should assist healthcare providers in understanding the impacts of the COVID‐19 outbreak on sleep.

## 2 METHODS

This systematic review is reported based on the Preferred Reporting Items for Systematic Reviews and Meta‐Analyses (PRISMA) guideline (Moher et al., 2010), a systematic literature search was done in five academic databases, relevant studies were abstracted, and their methodological quality was assessed using the Newcastle–Ottawa Scale (NOS) checklist. Findings were synthesised using a meta‐analysis approach. Results of the present paper are part of the findings from a larger project, the protocol of this project was registered in the International Prospective Register of Systematic Reviews (PROSPERO; identification code CRD42020181644) (Alimoradi & Pakpour, 2020).

### 2.1 Search strategy

Five academic databases including Scopus, PubMed Central, ProQuest, Web of Science (WoS), and the Excerpta Medica dataBASE (EMBASE) were searched systematically. The search terms were extracted from published reviews and primary studies besides PubMed Medical Subject Headings (MeSH). Specifically, the Patient‐problem, Exposure, Comparison, and Outcome (PECO) framework was used to determine search terms. In this regard, the “patient‐problem” was any human population, the “exposure” was COVID‐19 pandemic with a variety of factors contributing to sleep problems (including stress, reduced light exposure, extended working hours, and changed lifestyle), the “comparison” was none given that all the populations were impacted by exposure to the COVID‐19 pandemic, and the “outcome” was sleep. The main search terms were sleep and COVID‐19. The Boolean search method (AND/OR/NOT) was used to develop the search query. Search syntax was customised based on the advanced search attributes of each database. The search strategy is provided as Additional File 1. Additionally, reference lists of included studies were searched to increase the likelihood of retrieving relevant empirical studies.

### 2.2 Inclusion criteria

Observational studies, including case‐control and cross‐sectional studies, were included if relevant data relationships were reported. English, peer‐reviewed papers published between December 2019 and February 2021 were included. However, the papers were further screened to ensure that the data collection period was during the COVID‐19 pandemic or COVID‐19 endemic in mainland China. No limitation was imposed regarding participants characteristics. Sleep problems as primary outcomes should have been assessed using valid and reliable scales. Specifically, sleep problems defined in the present review are insomnia symptoms (assessed using Insomnia Severity Index [ISI] and Athens Insomnia Scale [AIS]) and poor sleep quality (assessed using Pittsburgh Sleep Quality Index [PSQI]).

#### 2.2.1 Primary outcome

Gender‐specific estimation of sleep problems prevalence during the COVID‐19 pandemic was the primary outcome.

#### 2.2.2 Secondary outcomes

1. Assessing the heterogeneity and its possible sources.
2. Influencing variables (e.g. age and marital status) in gender‐specific sleep problems prevalence during the COVID‐19 pandemic.

### 2.3 Study screening and selection

In the first step, the title and abstract of all retrieved papers were screened based on the inclusion criteria. The full texts of potentially relevant studies were further examined based on the aforementioned criteria. In this process, relevant studies were selected.

### 2.4 Quality assessment

The NOS was used to evaluate the methodological quality of the studies in observational studies. Three characteristics of selection, comparability, and outcome are examined with the NOS checklist. The checklist has three versions for evaluating cross‐sectional studies (seven items), case‐control (eight items), and cohort (eight items). Despite a slight difference in number and content of items, each item is rated with a star, except the comparability that can have two stars, thus resulting in a maximum score of 9. Studies with <5 points are classified as having a high risk of bias (Luchini et al., 2017). No studies were excluded based on the quality. But subgroup analysis was conducted to assess the impact of quality on pooled effect size.

### 2.5 Data extraction

A pre‐designed form was prepared to extract data from included studies. Data including first author’s name, collection date, study design, country, number of participants, gender, mean age, scale used to assess sleep problems, numerical results regarding the frequency of sleep problems. In the process of data extraction, two Excel sheets were initially designed, with one summarising the features of the included studies (e.g. author name and publication year) and the other evaluating methodological quality. The required data from the articles were later entered into another Excel datasheet for coding and preparing for analysis using STATA statistical software.

It should be noted that study selection, quality assessment, and data extraction were processes performed independently by two reviewers. In whole processes (i.e. study selection, quality assessment, and data extraction) disagreements were resolved through discussion by two independent reviewers. A third party was not required to resolve disagreements between the two independent reviewers because there were only minor disagreements, and both reviewers easily reached a consensus.

### 2.6 Data synthesis

A quantitative synthesis using STATA software version 14 was conducted. Meta‐analysis was run using a random effect model, as it was proposed that included studies were taken from different populations both within‐ and between‐study variances should be accounted for (Hox & Leeuw, 2003). The Q Cochrane statistic was used to assess heterogeneity. Also, the severity of heterogeneity was estimated using the *I* ^2^ index. Heterogeneity is interpreted as mild when *I* ^2^ is <25% and is considered moderate when *I* ^2^ is 25%–50%, and severe heterogeneity is diagnosed when *I* ^2^ is 50%–75%. An *I* ^2^ >75% is considered to have very severe heterogeneity (Huedo‐Medina et al., 2006).

Prevalence of sleep problems was the selected key measure for the present study. This pooled estimate of this key measure with 95% confidence interval (CI) is reported. Subgroup analysis or meta‐regression was done to find possible sources of heterogeneity and influencing variables on gender‐specific sleep problems prevalence. Funnel plot and the Begg's test were used to assess publication bias (Rothstein et al., 2005). Potential publication bias was corrected with the “fill‐and‐trim” method (Duval & Tweedie, 2000). The “Jackknife” method was used for sensitivity analysis (Hedges & Olkin, 2014).

## 3 RESULTS

### 3.1 Study screening and selection process

**FIGURE 1:**
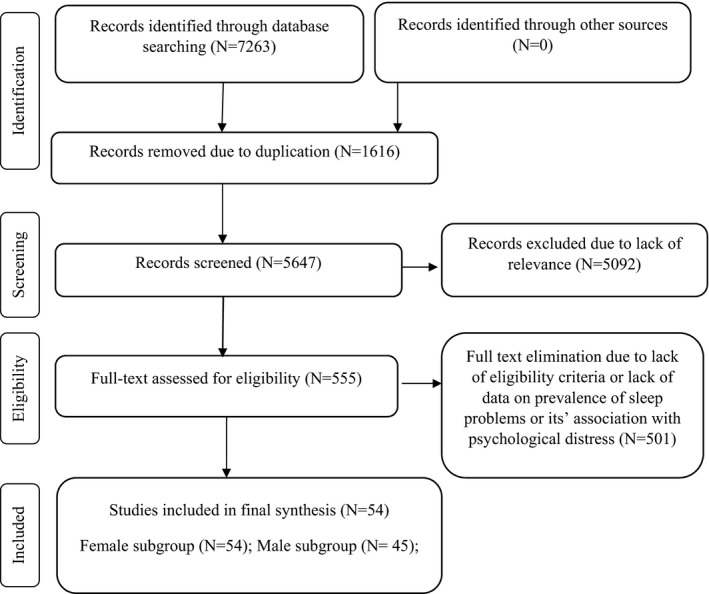
Search process based on the Preferred Reporting Items for Systematic Reviews and Meta‐Analyses (PRISMA) flowchart

The initial search of the five databases resulted in 7,263 studies: Scopus (*n* = 2,518), WoS (*n* = 474), PubMed (*n* = 338), EMBASE (*n* = 1,426), and ProQuest (*n* = 2,507). After removing duplicate papers, a further 5,647 papers were screened based on title and abstract. Finally, 555 papers appeared to be potentially eligible and their full texts were reviewed. In this process, 54 studies in the female subgroup and 45 studies in the male subgroup met the eligibility criteria and were pooled in the meta‐analysis. Figure 1 shows the search process based on the PRISMA flowchart.

## 4 MALE SUBGROUP

### 4.1 Study description

A total of 45 papers with 45,718 participants from 13 countries (China [38,545 participants], Italy [2717], Austria [475], Turkey [480], Bangladesh [223], Pakistan [406], Greece [40], India [340], Belgium, [81] Egypt [133], Saudi Arabia [295], UK [45], and Iran [1,314]) were included. Four papers gathered data during the lockdown period. The smallest sample size was 12, and the largest was 27,149. The individual country with the most eligible studies was China (*N* = 23). The mean age of participants varied from 15.5 to 70 years and ~65.9% were married. Most studies involved the general population (24 studies), with others involving health professionals (14), and patients with COVID‐19 (seven). Most of the studies were cross‐sectional (43 studies). The two remaining studies had a longitudinal design and collected data during the COVID‐19 pandemic and baseline data were extracted. The ISI and PSQI were used to assess sleep problems (in 25 and 14 studies, respectively). Considering NOS >5 as high quality, 71% of the included studies (32 papers) were categorised as high‐quality. Table 1 provides the summary characteristics of the included studies.

**TABLE 1:** Summarised characteristics of included studies

ID	Authors	Year	Country	Collection date	Lockdown period	Design	Participant group	Sample size, *n*	Sex, % female	% Married	Age, years mean/range	NOS	Sleep problem scale
3	Zhang (Zhang, Yang et al., 2020)	2020	China	January 29–February 3, 2020	no	cross‐sectional	medical staff	1,563	82.73	63.92	18–>60	5	ISI
5	Huang (Huang & Zhao, 2020)	2020	China	February 3–10, 2020	no	cross‐sectional	volunteer population	603	69		36.5	5	PSQI
28	Fu (Fu et al., 2020)	2020	China	February 18–28, 2020	no	cross‐sectional	Wuhan residents	1,242	69.73	33.7	>18	5	AIS
30	Zhang (Zhang, Zhang et al., 2020)	2020	China	February 19–March 20, 2020	no	longitudinal surveys	college students	66	62.12		20.70	5	PSQI
32	Li (Zhou, Shi et al., 2020)	2020	China	April 25–May 9, 2020	no	cross‐sectional	workers with income losses	398	49.5	49.5	18–>40	9	ISI
34	Wang (Wang, Xie et al., 2020)	2020	China	January 30–February 7, 2020	no	cross‐sectional	medical workers	123	90	30.08	33.75	6	PSQI
35	Hu (Giardino et al., 2020)	2020	China	March 7–24, 2020	no	cross‐sectional	COVID−19 inpatients	85	49.4	85.9	48.8	6	ISI
36	Yang (Xiao et al., 2020)	2020	China	March 5–14, 2020	no	cross‐sectional	general population	2,410	49.2	76.55	36.3	5	PSQI
45	Gualano (Gualano et al., 2020)	2020	Italy	April 19 and May 3, 2020	yes	cross‐sectional	general population	1,515	65.6	61.1	42	5	ISI
57	Pieh (Pieh et al., 2020)	2020	Austria	April 15–30, 2020	yes	cross‐sectional	general population	1,005	52.7		18–>65	6	ISI
65	Zhuo (Zhuo et al., 2020)	2020	China	March 2020	no	cross‐sectional	medical staff	26	46.15		41.92	5	ISI
69	Wang (Ren et al., 2020)	2020	China	February 2 and 3, 2020	no	cross‐sectional	medical staff	1,045	85.8			7	ISI
70	Shi (Shi et al., 2020)	2020	China	February 28–March 11, 2020	no	cross‐sectional	general population	56,932	52.1	77.2	35.97	7	ISI
11	Lai (Lai, Ma et al., 2020)	2020	China	January 29–February 3, 2020	no	cross‐sectional	healthcare workers	1,257	76.7	66.7	18–>40	6	ISI
46	Zhou (Zhou, Yang et al., 2020)	2020	China	March 24–3 April, 2020	no	cross‐sectional	healthcare workers	1,931	95.4	63.4	35.08	5	PSQI
56	Zhang (Zhang, Xu et al., 2020)	2020	China	January 25 and March 15	no	retrospective cohort	Covid‐19 patients	136	42.2	95.6	63	6	PSQI
554	Wasim (Wasim et al., 2020)	2020	Pakistan	May 20–June 3, 2020	no	cross‐sectional	tertiary care hospital dealing with corona patients	356	52.00	51.40	20–>50	6	ISI
537	Sharma (Sharma et al., 2020)	2020	India	0	no	cross‐sectional	obstetrics staff	184	58.70	54.35	20–>50	5	ISI
535	Tiete (Tiete et al., 2020)	2021	Belgium	April 17–May 25, 2020	no	cross‐sectional	healthcare professionals	647	78.40	80.50	20–>50	8	ISI
511	Franceschini (Franceschini et al., 2020)	2020	Italy	March 10–May 4, 2020	yes	cross‐sectional	general population	6,439	73.10	65.10	33.90	6	Medical Outcomes Study–Sleep Scale (MOS‐SS)
447	Bhat (Bhat et al., 2020)	2020	Kashmir	April 4–10, 2020	no	cross‐sectional	general population	264	27.70		<18–>60	8	PSQI
420	Liu (Liu et al., 2020)	2021	China	February 1–10, 2020	no	cross‐sectional	general population	2,858	53.60	60.20	<18–>50	6	PSQI
410	Alamrawy (Alamrawy et al., 2021)	2021	Egypt	July 2–23, 2020	no	cross‐sectional	young adults of both genders aged between 14 and 24 years	447	70.20		20.72	8	ISI
397	Akıncı (Akıncı & Başar, 2021)	2021	Turkey	April and May, 2020	no	cross‐sectional	patients hospitalised with COVID‐19	189	41	82.50	46.27	6	PSQI
394	Barua (Barua et al., 2020)	2021	Bangladesh	April 1–May 30, 2020	no	cross‐sectional	healthcare professionals	370	39.70	66.80	30.50	8	SCI‐02
389	Fidanci (Fidanci et al., 2020)	2020	Turkey	May 2020	no	cross‐sectional	healthcare professionals	153	67.30		33.40	5	PSQI
376	Gu (Peng et al., 2020)	2020	China	February 15–22, 2020	no	cross‐sectional	patients with COVID‐19	461	64.90	95.90	18–>50	5	ISI
348	Almater (Almater et al., 2020)	2020	Saudi Arabia	March 28–April 4, 2020	no	cross‐sectional	ophthalmologists	107	43.90		32.90	8	ISI
12	Khoury (Khoury et al., 2021)	2021	Canada	June 3 and July 31, 2020	no	cross‐sectional	pregnant individuals	303	100.00	100.00	32.13	7	ISI
17	Wang (Wang, Zhao et al., 2020)	2021	China	January 28–March 31, 2020	no	cross‐sectional	general population	5,676	71.40	68.90		6	ISI
25	Zreik (Zreik et al., 2021)	2021	Israel	20–30 April, 2020	yes	cross‐sectional	general population	264	100	100	33.97	5	ISI
47	Xie (Xie et al., 2021)	2020	China	0	no	cross‐sectional	pregnant individuals	689	100	100	29.03	6	PSQI
48	Zhang (Zhang et al., 2021)	2021	China	January–February, 2020	no	cross‐sectional	pregnant individuals	456	100	100		6	PSQI
57	Massicotte (Massicotte et al., 2021)	2021	Canada	April 28 and May 29, 2020	no	cross‐sectional	breast cancer patients	36	100	66.7	53.6	5	ISI
67	Chen (Chen, Wang et al., 2021)	2021	China	March 14–21, 2020	no	cross‐sectional	breast cancer patients	834	100	86		5	ISI
81	Yadav (Yadav et al., 2021)	2021	India	June–August, 2020	no	cross‐sectional	COVID‐19 patients	100	27		42.9	5	ISI
92	Bacaro (Bacaro et al., 2020)	2020	Italy	April 1– May 4, 2020	yes	cross‐sectional	general population	1,989	76.17		38.4	7	ISI
106	Zhou (Zhou, Shi et al., 2020)	2020	China	February 28–March 12, 2020	no	cross‐sectional	general population of pregnant and non‐pregnant women	859	100	93.25	33.25	9	ISI
120	Fazeli (Fazeli et al., 2020)	2020	Iran	May 2–August 26, 2020	no	cross‐sectional	adolescents	1,512	43.6		15.51	9	ISI
130	Şahin (Şahin et al., 2020)	2020	Turkey	April 23 and May 23, 2020	no	cross‐sectional	healthcare workers	939	66	65.7	18–>40	9	ISI
137	Lai (Lai, Lee et al., 2020)	2020	UK	April 28–May 12, 2020	no	cross‐sectional	international university students	124	63.7			9	ISI
138	Wang (Wang, Chen et al., 2020)	2020	China	February 21–March 7, 2020	no	cross‐sectional	college students	3,092	66.4			9	SRSS
159	Wang (Wang, Zhu et al., 2020)	2020	China	March 2020	no	cross‐sectional	COVID‐19 inpatients	484	50.2	91.7	52.5	9	ISI
164	Xia (Xia et al., 2020)	2020	China	April 20–30, 2020	no	case‐ control	patients with Parkinson disease	288	51.85		60.50	9	PSQI
174	Alnofaiey (Alnofaiey et al., 2020)	2020	Saudi Arabia	May–August, 2020	no	cross‐sectional	healthcare workers	340	49.1		20–60	9	PSQI
190	Juanjuan (Juanjuan et al., 2020)	2020	China	February 16–19, 2020	no	cross‐sectional	patients with breast cancer	658	100	88.9		9	ISI
201	Wang (Wang, Gong et al., 2020)	2020	China	February 4–18, 2020	no	cross‐sectional	general population	6,437	56.13	38.99		9	PSQI
277	Parlapani (Parlapani et al., 2020)	2020	Greece	0	no	cross‐sectional	general population	103	61.17		69.85	9	AIS
239	Lin (Chang et al., 2020)	2020	Iran	February 15–30, 2020	no	cross‐sectional	general population	1,078	58.3		26.24	9	ISI
375	Ahorsu (Ahorsu, Lin, & Pakpour, 2020)	2020	Iran	April 1–30, 2020	no	cross‐sectional	general population	413	38	87.9	57.72	9	ISI

Abbreviations: AIS, Athens Insomnia Scale; COVID‐19, coronavirus disease 2019; ISI, Insomnia Severity Index; NOS, Newcastle–Ottawa Scale; PSQI, Pittsburgh Sleep Quality Index; SCI‐02, Sleep Condition Indicator two‐item short‐form; SRSS, Self‐Rating Scale of Sleep.

### 4.2 Estimation of sleep problem prevalence

**FIGURE 2:**
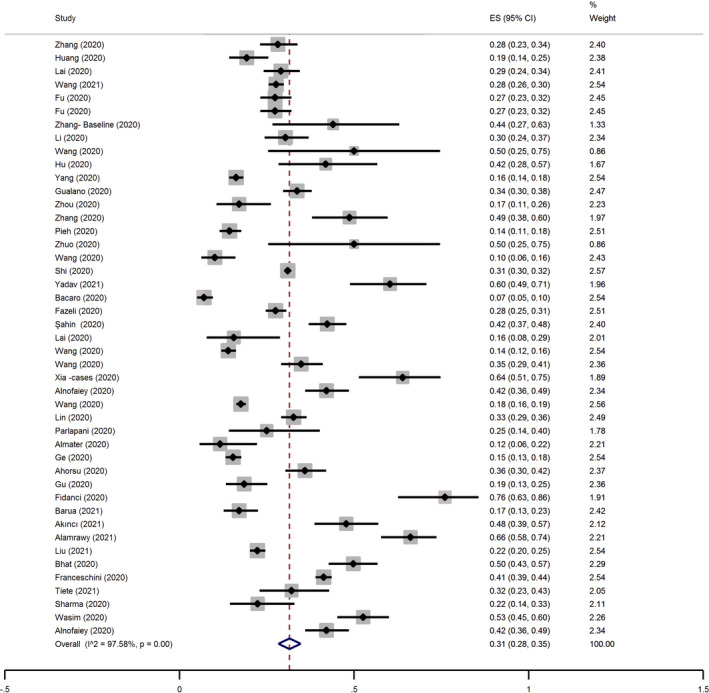
Forest plot for the pooled prevalence of sleep problems in the male group. CI, confidence interval; ES, effect size

The pooled estimated prevalence of sleep problems was 31% (95% CI 28%–35%; *I* ^2^: 97.58%, tau^2^: 0.01). Figure 2 provides a Forest plot of the pooled prevalence of sleep problems in this group.

Subgroup analysis (Table 2) showed that the prevalence of sleep problems was higher in longitudinal versus cross‐sectional studies (48% versus 31%). Although prevalence of sleep problems appeared to be different among male healthcare professionals (34%), the general population (29%) and patients with COVID‐19 (39%), these differences were not statistically significant considering overlap in the 95% CI of pooled prevalence among these groups (26%–43% for healthcare professionals, 24%–33% for general population, and 27%–50% for patients with COVID‐19). Based on multivariable meta‐regression (Table 4), being in lockdown period, quality of studies, and measure used to assess sleep problems were significant predictors of sleep problems prevalence among male participants. These variables together explained 100% of the variance.

**TABLE 2:** Results of subgroup analysis for estimated pooled prevalence

Variable	Female participants (*N* = 54 studies)			Male participants (*N* = 45 studies)		
	No. studies	Pooled prevalence, % (95% CI)	*I* ^2^, %	No. studies	Pooled prevalence, % (95% CI)	*I* ^2^, %
Lockdown period						
Yes	5	37 (13–62)	99.83	4	24 (6–42)	99.4
No	49	41 (36–45)	99.24	41	32 (29–35)	96.7
Study quality						
Low quality	16	38 (31–45)	98.27	13	32 (25–38)	93.95
High quality	38	41 (36–47)	99.56	32	31 (28–35)	97.96
Study design						
Cross sectional	52	40 (35–45)	99.45	45	31 (28–34)	97.6
Longitudinal	2	55 (46–65)	‐	2	48 (38–57)	‐
Participants’ group						
Health professionals	15	41 (31–51)	99.02	15	34 (26–43)	94.8
General patients	32	38 (32–44)	99.58	25	29 (24–33)	98.4
COVID‐19 patients	7	51 (42–60)	84.68	7	39 (27–50)	91.3
Measure of Sleep						
ISI	31	41 (36‐47)	99.33	14	30 (26–34)	96.7
PSQI	17	41 (33–50)	99.08	27	38 (31–44)	97.2
Other	6	34 (13–55)	99.76	6	25 (14–37)	98.4
Overall estimated prevalence	54	41 (37–46)	99.41	45	31 (25–38)	97.48

Abbreviations: COVID‐19, coronavirus disease 2019; ISI, Insomnia Severity Index; PSQI, Pittsburgh Sleep Quality Index.

**FIGURE 3:**
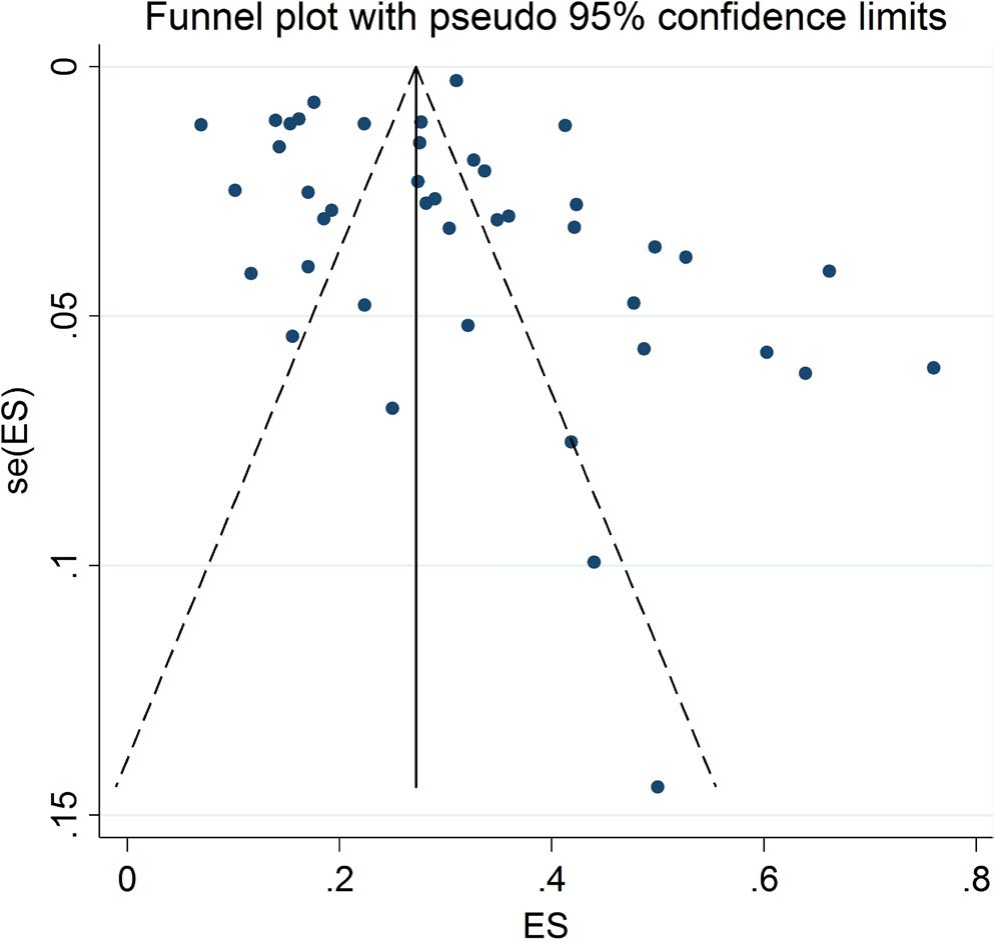
Funnel plot assessing the publication bias among the included studies in the male subgroup. ES, effect size

**FIGURE 4:**
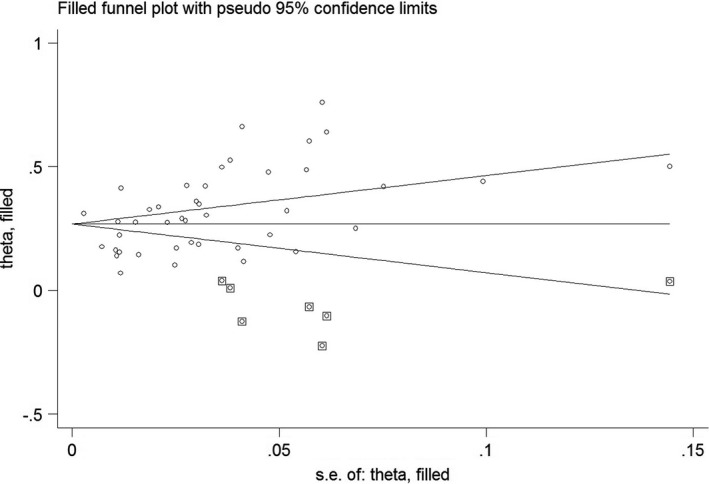
Corrected funnel plot based on the fill‐and‐trim method in the male subgroup

Begg’s test (*p* = 0.006) and funnel plot (Figure 3) consider probability of publication bias. Meta trim was used to correct publication bias. Based on the trim method, eight studies were imputed, and the corrected prevalence of sleep problems was 27% (95% CI 24%–30%). The corrected funnel plot is provided in Figure 4. Also, sensitivity analysis showed that pooled effect size was not affected by the effect of a single study.

## 5 FEMALE SUBGROUP

### 5.1 Study description

A total of 54 papers with 67,722 participants from 15 countries (China [54,801 participants], Italy [7,222], Austria [530], Turkey [801], Bangladesh [147], Pakistan [907], Greece [63], India [12,266], Belgium [507], Egypt [314], Saudi Arabia [274], UK [79], Canada [339], Israel [264], and Iran [12,266]) were included. Five papers gathered data during the lockdown period. The individual country with the most eligible studies was China (*N* = 29). The smallest sample size was 14, and the highest was 29,530. The mean age of participants varied from 15.4 to 70 years and ~72.1% were married. Most studies involved the general population (32 studies), with others involving health professionals (15), and patients with COVID‐19 (seven). Most of the studies were cross‐sectional (52 studies). The two remaining studies had a longitudinal design and collected data during the COVID‐19 pandemic and baseline data were extracted. The ISI and PSQI were used to assess sleep problems (in 31 and 17 studies, respectively). Considering NOS >5 as high quality, 70% of the included studies (38 papers) were categorised as high‐quality. Table 1 provides the summary characteristics of the included studies.

### 5.2 Estimation of sleep problem prevalence

**FIGURE 5:**
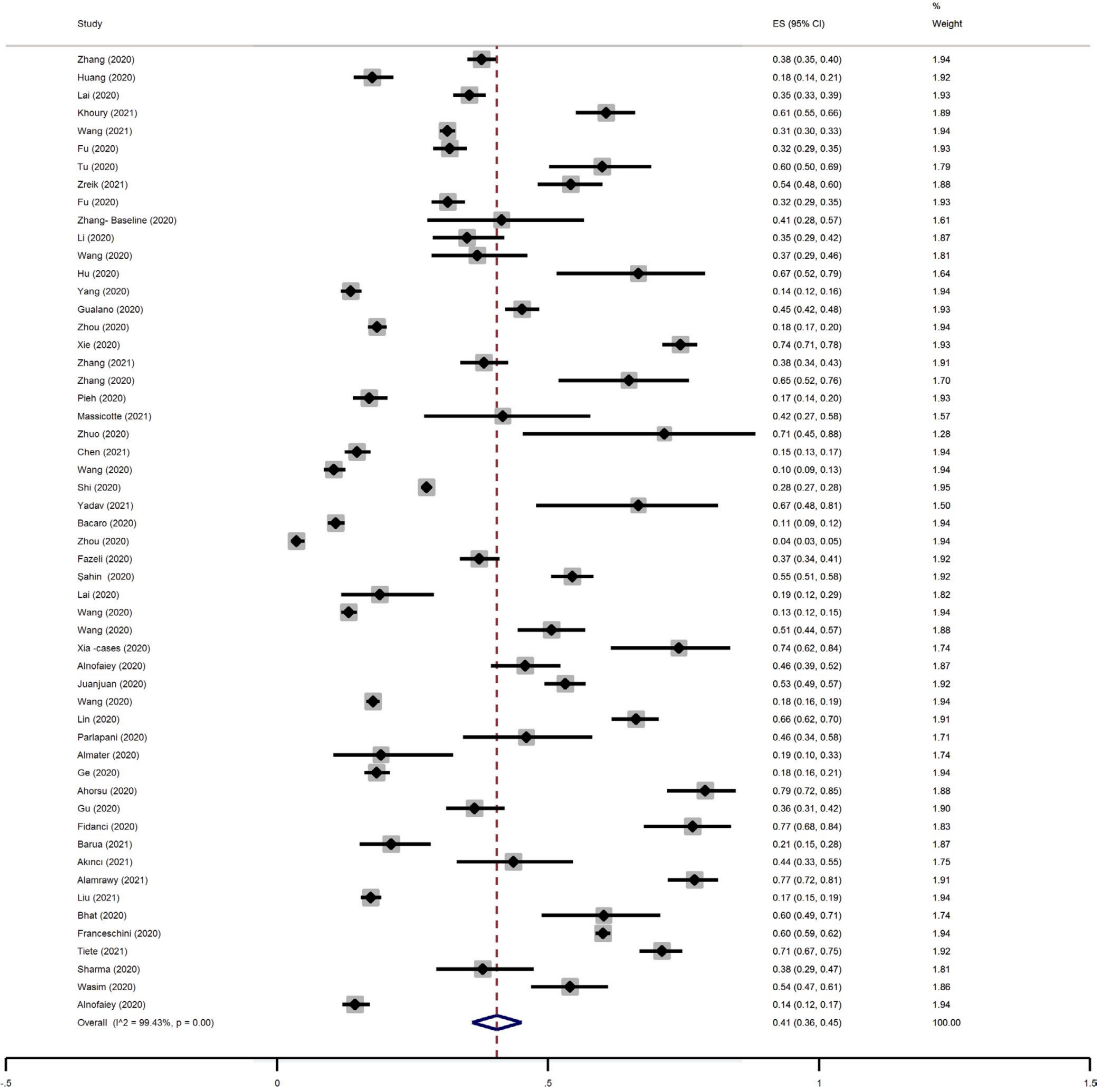
Forest plot for the pooled prevalence of sleep problems in the female group. CI, confidence interval; ES, effect size

The pooled estimated prevalence of sleep problems was 41% (95% CI 36%–45%; *I* ^2^: 99.43%, tau^2^: 0.03). Figure 5 provides a Forest plot regarding the pooled prevalence of sleep problems in this group.

Subgroup analysis (Table 2) showed that the prevalence of sleep problems was higher in longitudinal versus cross sectional studies (55% versus 41%). Although prevalence of sleep problems appeared to be different among female patients with COVID‐19 (51%), healthcare professionals (41%), and the general population (38%), these differences were not significantly different considering the overlap in 95% CI of pooled prevalence among these groups (31%–51% for healthcare professionals, 32%–44% for general population, and 42%–60% for patients with COVID‐19). Based on univariate meta‐regression (Table 3), country and percentage of married participants were other significant predictors of sleep problems prevalence among women. In multivariable meta‐regression (Table 4) being in lockdown and study quality were significant predictors of sleep problems prevalence among female participants, which explained 34.18% of the variance.

**TABLE 3:** Results of meta‐ regression for gender‐specific estimated pooled prevalence

Univariable	Female							Male						
Variable	No. studies	Coeff.	SE	*p*	*I* ^2^ res., %	Adj. *R* ^2^, %	Tau^2^	No. studies	Coeff.	SE	*p*	*I* ^2^ res., %	Adj. *R* ^2^, %	Tau^2^
Country	54	0.01	0.003	0.02	99.35	9.26	0.04	45	0.002	0.002	0.35	97.59	0.51	0.02
Age	30	0.004	0.003	0.29	99.59	0.44	0.05	24	0.001	0.003	0.62	97.75	−3.32	0.03
% of married participants	34	0.0003	0.002	0.05	99.53	8.51	0.03	25	0.001	0.001	0.43	95.87	−0.88	0.01

Abbreviation: Coeff., coefficient.

**TABLE 4:** Results of multivariable meta‐regression for estimated pooled prevalence

Variable	Female participants			Male participants		
	Coefficient	SE	*p*	Coefficient	SE	*p*
Country	0.007	0.004	0.14	−0.002	0.001	0.32
Design	−0.07	0.13	0.59	−0.02	0.04	0.57
Lockdown period (yes versus no)	0.41	0.16	0.03	0.19	0.04	0.02
Study quality (low versus high quality)	0.34	0.12	0.02	0.23	0.03	0.004
Participants group	−0.03	0.07	0.73	0.007	0.01	0.64
Age	0.009	0.006	0.15	0.007	0.002	0.06
% married of participants	0.002	0.003	0.52	−0.003	0.002	0.20
Measure of sleep	−0.16	0.09	0.09	−0.11	0.03	0.04
Number of included studies in multivariable regression	18			12		
Between‐study variance (tau^2^)	0.03			0.004		
% residual variation due to heterogeneity (*I* ^2^ residual)	98.98			0		
Proportion of between‐study variance explained (Adjusted *R* ^2^)	34.18			100		

**FIGURE 6:**
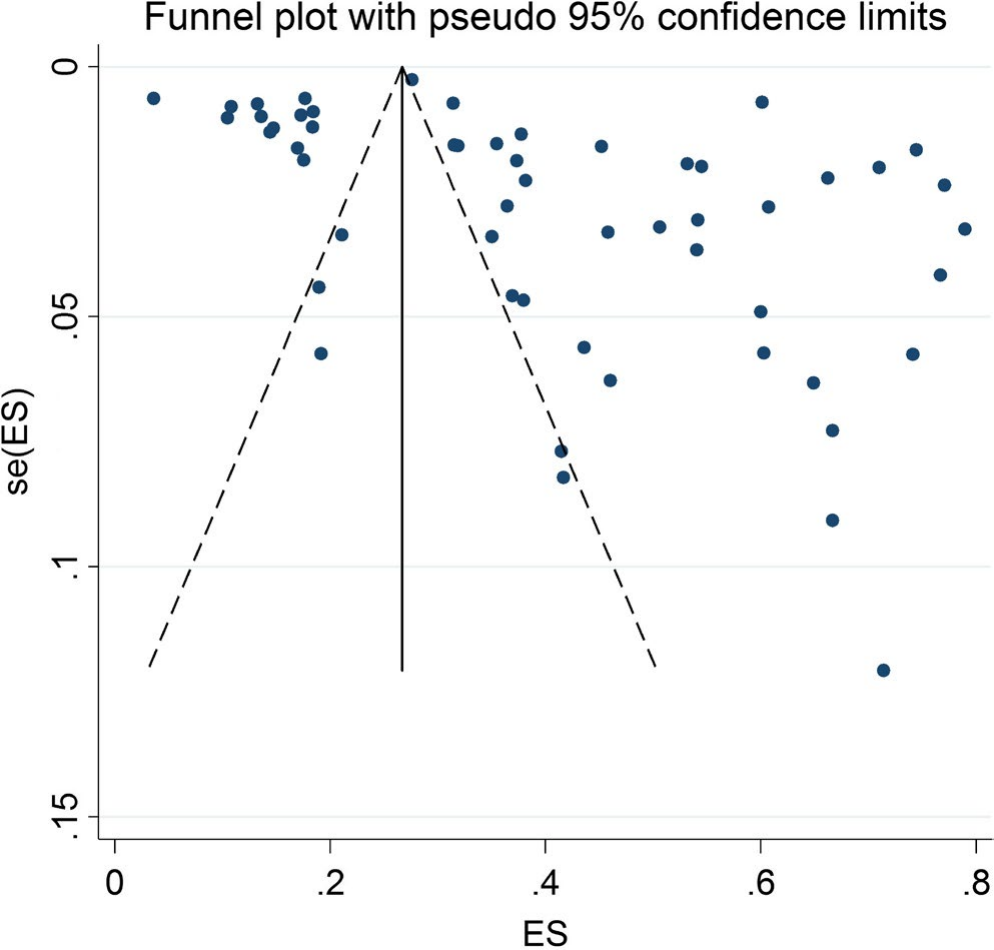
Funnel plot assessing the publication bias among included studies in the female subgroup. ES, effect size

**FIGURE 7:**
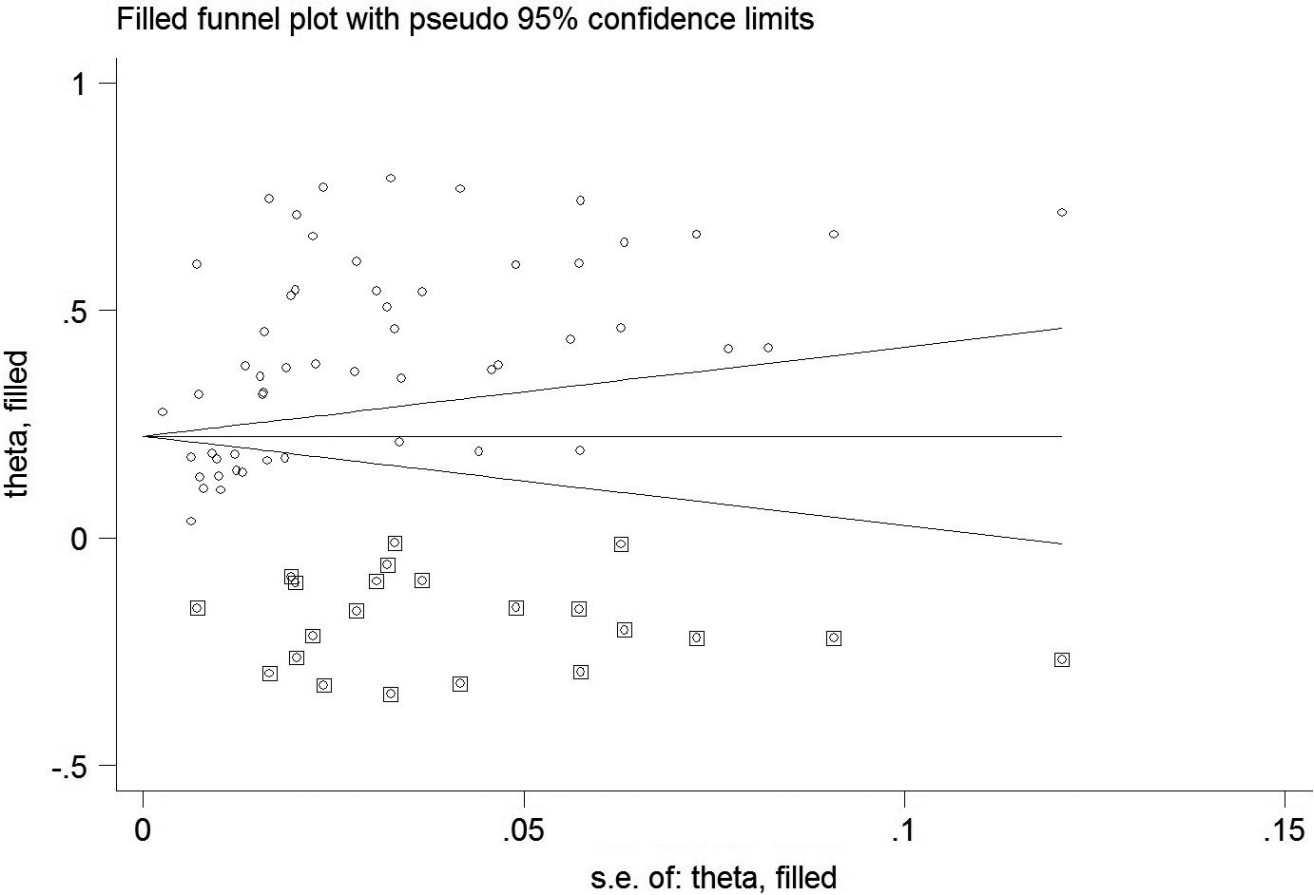
Corrected funnel plot based on the fill‐and‐trim method in the male subgroup

As indicated above, the Begg’s test (*p* = 0.08) and funnel plot (Figure 6) consider probability of publication bias. Meta trim was used to correct publication bias. Based on the trim method, 22 studies were imputed, and the corrected prevalence of sleep problems was 24% (95% CI 19%–29%). The corrected funnel plot is provided in Figure 7. Also, sensitivity analysis showed that the pooled effect size was not affected by the effect of a single study.

## 6 DISCUSSION

The present systematic review and meta‐analysis aimed to provide timely information for healthcare providers to understand how the COVID‐19 pandemic and the related government actions impacted on sleep problems worldwide. More specifically, the present study estimated the prevalence of sleep problems separately for males and females using amalgamated data from 54 recently published studies in the female subgroup and 45 recently published studies in the male subgroup. With the use of the PRISMA guideline and rigorous meta‐analysis methods, robust and valid information on the prevalence of sleep problems between males and females worldwide are provided in the present study. We should note that the estimate of sleep problems was calculated based on the reports emanating from 15 countries for the female subgroup and 13 countries for the male subgroup with nearly 115,000 participants, and therefore, expanded information originating from other regions would be valuable to assess for the consistency and applicability of the present findings. As a corollary to these considerations, we uncovered sex differences in the prevalence of reported sleep problems with women exhibiting greater prevalence. Moreover, subgroup analysis and meta‐regression showed a lower rate of prevalence for sleep problems regardless of gender in regions where the lockdown was implemented than in regions where control measures without lockdown were put in place. Additionally, COVID‐19‐infected patients had higher prevalence rates of sleep problems than did health professionals and the general population. It is possible that such effects of COVID‐19 reflect central nervous system involvement by the virus or unspecific consequences of the disease stress induced by the infection (Cénat et al., 2020). Notwithstanding, female health professionals appear to be more likely to experience sleep problems compared to their counterparts in the general population, but such differences did not emerge in men.

As indicated, most of the data retrieved for the present systematic review and meta‐analysis originated from cross‐sectional designed studies. Notwithstanding, we surmise that the fear and stress associated with COVID‐19 may be one of the major reasons contributing to the high prevalence of sleep problems. More specifically, social media and news channels have continuously routinely reported on daily deaths and on the number of cumulative infected cases of COVID‐19 both at the national and global scales, and such intensive media exposure is likely to generate the anxiety and stress that facilitate the emergence of sleep problems (Lin, Broström et al., 2020, Lin, Imani et al., 2020). Indeed, higher levels of psychological distress and signs of mental disorders have been reported during this pandemic among different populations worldwide (Mamun et al., 2021; Rodríguez‐Rey et al., 2020; Wang, Pan et al., 2020) and significant sleep difficulties have been identified in the context of major public health threats (e.g. Ebola) (Cates et al., 2018; Lehmann et al., 2015).

The reasons for the higher prevalence of sleep problems in females are unclear, but possibly may reside in the underlying brain structural differences across sexes (Xin et al., 2019). Therefore, exposure to the same circumstances may yield different perceptions and lead to divergent emotional processing. Indeed, prior evidence found that self‐reported outcomes on subjective health (e.g. quality of life) differ between males and females (Lin et al., 2016; Su et al., 2013). Additionally, women are more likely to report psychological problems in response to taxing situational settings (Wang et al., 2017). Finally, issues such as insomnia exhibit clear gender dimorphic features (Kocevska et al., 2020; Silva‐Costa et al., 2020; Sivertsen et al., 2021).

The sleep problems among healthcare professionals found in the present systematic review and meta‐analysis could be attributed to the interactions between the COVID‐19 pandemic and the specific attributes of the jobs. From the perspective of the COVID‐19 pandemic, health professionals, especially those who had to be in direct contact with patients with COVID‐19 and those who were at high risk of being exposed to the COVID‐19 virus, had higher levels of worry and psychological distress. The higher levels of worry and psychological distress are likely to subsequently foster the development of their sleep problems (Fidanci, derinöz Güleryüz, & Fidanci, 2020). From the perspective of the job itself, health professionals, especially those who work in a large hospital, have irregular work schedules when compared to individuals who work in other occupations (Caruso, 2014; Ferri et al., 2016; Jahrami et al., 2019; Koinis et al., 2015; Kumar et al., 2018; Mohanty et al., 2019). Such irregular work schedules are harmful for a good night’s sleep. Therefore, the interaction between the COVID‐19 pandemic and job type may increase the workload for healthcare professionals and exacerbate their sleep issues.

There are some strengths and limitations of the present study that deserve mention. First, the timely and comprehensive search of the literature ensures that the information and estimates reported reflect the available state of knowledge. Moreover, inclusion of different cohorts such as those represented by patients with COVID‐19, healthcare professionals, and the general populations provide a wider perspective on the effects of the pandemic on sleep. Second, the present systematic review and meta‐analysis utilised robust and rigorous methodology to ensure the quality of the analysed studies and synthesised estimations. More specifically, the literature search was systematically conducted in several major databases, including Scopus, PubMed Central, ProQuest, ISI WoS, and EMBASE. All the review processes were conducted using the international standard, i.e. PRISMA guidelines (Moher et al., 2010), and the NOS checklist was used to ascertain the quality of each study. Third, the cumulative sample size was relatively large (>100,000) and encompassed 15 countries (China, Italy, Austria, Turkey, Bangladesh, Pakistan, Greek, India, Belgium, Egypt, Saudi Arabia, UK Canada, Israel, and Iran), likely adding generalisability to the findings of the present study. However, we should also point out that a cross‐sectional design was the most used design among the included papers, and thus the causal relationship between the COVID‐19 outbreak and sleep problems is tentative at best. More specifically, it is unclear whether the prevalence of sleep problems was significantly changed between *before* and *during* the COVID‐19 outbreak. Furthermore, sleep problems estimates were derived from different survey instruments, which obviously differ in their psychometric properties and may also differentially capture heterogeneous aspects of sleep problems. More specifically, some people may be for example identified as having sleep problems using the ISI, but not with the PSQI. Therefore, the biases in estimating prevalence of sleep problems cannot be overcome. Third, the measures used to identify sleep problems were all based on self‐reporting. Therefore, commonly encountered biases (e.g. recall bias and social desirability bias) cannot not be excluded. Fourth, the actual figures of COVID‐19 regarding suspected cases, confirmed cases, and deaths are widely different across countries; therefore, the impact of such figures on sleep problems may not be the same. Furthermore, different countries applied different policies for COVID‐19 outbreak control (Chang et al., 2020; Chen, Chen et al., 2021, Chen, Wang et al., 2021; Chen et al., 2020; Lin, Broström et al., 2020, Lin, Imani 2020; Mamun et al., 2021; Pramukti et al., 2020) and such measures could affect the prevalence rates of sleep problems.

In summary, a relatively high prevalence of sleep problems emerged during the COVID‐19 pandemic and imposed increased effects on women. The sleep problems found in the present systematic review and meta‐analysis concur with the evidence of well‐established adverse impacts of long‐term lockdown on mental health (Ahorsu, Lin, & Pakpour, 2020; Chang et al., 2020; Lin, Broström et al., 2020, Lin, Imani et al., 2020). Considering the present findings, specific measures aimed at mitigating the effect of the COVID‐19 pandemic on sleep should be developed and tried in a gender‐specific fashion.

## AUTHOR CONTRIBUTIONS

Each author made a substantial contribution to project design, data collection or data analysis. Additionally, all authors contributed to the preparation of this manuscript.

## CONFLICT OF INTEREST

All authors have no conflicts to declare.

## FUNDING INFORMATION

The open access was funded by Jönköping University.

## Supporting information

Supplementary Material

## Data Availability

Data sharing not applicable to this article as no datasets were generated or analysed during the current study.
